# From Visual Attention to Literacy: Symbol Search Deficit Predicts Future Reading Difficulties From Age 3

**DOI:** 10.1111/desc.70258

**Published:** 2026-07-22

**Authors:** Audrey Vialatte, Eric Chabanat, Pierre‐Emmanuel Aguera, Agnes Witko, Laure Pisella

**Affiliations:** ^1^ Lyon Neuroscience Research Center INSERM U1028 CNRS UMR 5292, Claude Bernard Lyon1 University Bron Cedex France; ^2^ Institut des Sciences et Techniques de Réadaptation Claude Bernard, Lyon1 University Lyon France; ^3^ Laboratoire Dynamique du language CNRS UMR 5596, Université Lumière Lyon2 Lyon France

**Keywords:** developmental dyslexia, early visuo‐attentional predictor of reading, longitudinal study, parallel processing, preschool predictors, reading fluency, screening test, spatial binding, written symbols

## Abstract

Poor reading has been associated with impaired simultaneous visual processing of multiple graphic symbols composed of separable features, a behavioral marker of reduced visual attention based on parietal dysfunction. We designed a visual search screening test, consisting of finding the (unique) black circle among circles combined with a bar of various length and location (black symbols) versus among circles of various colors. The temporal Gap between these symbol and color search conditions was previously validated as a significant visuo‐attentional contributor of reading between ages 8 and 11, predicting its performance with moderate sensitivity, as reading difficulty is multifactorial, but high specificity. Here, we established its good positive predictive value in a longitudinal study with the same children being screened in kindergarten (at 3, 4, or 5 years old) and later assessed for reading performance at the second semester of third grade (at 8 years old). We also assessed 4 and/or 5 years old children with the knowledge of letter names, the early reading of classmates’ first names and of small frequent words. The visual search screening test predicted future reading performance with lower correlation strength but as early as 3 years old, providing more lead time for specific preventive educational or clinical intervention during kindergarten in a subgroup whose pathological visual search performance at Age 3 was associated with a 5.7 times greater likelihood of later reading difficulties.

## Summary

- Written symbols are specific visual stimuli made up of a spatial combination of strokes.
- Gap between search performance with symbols versus single‐unit objects reflects visual attention capacity for simultaneous processing.
- The Gap measure of visual search screening test can be used as early visuo‐attentional predictor of reading difficulty.
- Pathological performance at the visual search screening test at Age 3 predicted third‐grade reading efficiency with 36% sensitivity and 94% specificity.
- The good positive predictive value of our screening at Age 3 (62.5%) provides lead time for preventive program to improve visual attention for graphic symbols.

## 1 Introduction

### 1.1 General Context of the Ludisymboles Research Project: The Contribution of Visual Attention to Reading

Developmental dyslexia (DD) is defined as a persistent difficulty in acquiring fluent and automatic reading despite normal intelligence and adequate educational opportunities (Rutter and Yule 1975). As reading is intrinsically a visual process that requires efficient visual recognition and visuo‐attentional sampling of letters, a body of research indicates that deficits in visual attention (VA) are present in a subset of individuals with DD, with or without the association of phonological impairments (Lobier et al. 2012; Saksida et al. 2016; Valdois et al. 2011; Zoubrinetzky et al. 2014). However, the phonological deficit remains the most widely recognized—and often the sole—explanatory factor for DD (Hulme 1987; Goswami 2015). In her review article, Goswami (2015) highlighted the two main missing arguments for a VA theory of DD. The first was that it must explain how a sensory deficit will only manifest itself in “linguistic” situations and not in other everyday life situations. For example, phonology is considered as the auditory processes specific to the sounds of oral language (Serniclaes et al. 2001). The second was the lack of longitudinal studies demonstrating the causality of VA disorders by highlighting their presence before learning to read. For example, oral language measures (like syntactic and phonological accuracy in repeating sentences, words and syllables) can be administered as early as 2.5–3 years of age and reliably predict later reading outcomes (Scarborough 2001). No such VA predictor is available as early as Age 3, leading Goswami (2015) to conclude that VA deficits in DD could be solely ascribed to poor reading experience.

Developing a predictive measure capable of detecting VA vulnerabilities by Age 3 would therefore be highly valuable, both theoretically and practically. Indeed, such early identification provide teachers and/or clinicians with sufficient lead time to implement specific preventive pedagogical strategies and/or intensive training before the first grade of formal schooling. Establishing such a VA marker requires a longitudinal design in which the same children are assessed by Age 3 and later evaluated for reading outcomes, preferentially in third grade, as the transition from “learning to read” to “reading to learn” is typically completed by this stage, which allows the detection of durable reading acquisition delays (Aghababian and Nazir 2000; Chall 1983; LaBerge and Samuels 1974).

The ludisymboles project aimed to evaluate the predictive value of an early marker of the VA difficulties that could hinder reading automatization. It would enable to focus on strengthening the visual prerequisites of reading from Age 3, during kindergarten. Unlike specificity, sensitivity of such an early VA predicter of reading difficulties was neither expected nor aimed, first, because VA is not the sole factor contributing to reading acquisition and second, because early oral language predictors already exist to detect the reading difficulties caused by phonological deficits.

### 1.2 General Context of the Ludisymboles Research Project: Distal Early Predictors and Proximal Later Predictors of Reading Performance

The National Early Literacy Panel's (2008) meta‐analysis highlighted that the strongest predictors are phonological awareness, letter‐name and letter‐sound knowledge, as well as rapid automatized naming (of letters, numbers, colors, or pictures), the ability to write letters from dictation or to write one's own name. These predictors are proximal to reading and can be tested only at the end of kindergarten, as they involve letter knowledge, familiarity with the alphabetic code and left‐right horizontal scanning, in addition to VA.

Distal measures exist, that significantly predict later reading ability as early as 2.5–3 years of age, but they assess oral language difficulties and are therefore not specific to written language. In contrast, VA difficulties have been closely associated with bilateral dysfunction of the superior parietal lobule (SPL) rather than of the left‐hemispheric oral language network (Liu et al. 2022; Lobier et al. 2012, 2014; Lobier and Valdois 2015; Peyrin et al. 2011, 2012; Reilhac et al. 2013; Valdois et al. 2019a, 2021; Vialatte et al. 2021a, 2021b, 2023a). Non‐phonological difficulties correlated to reading performance are characterized as a reduced ability to process multiple alphabetic or non‐alphabetic symbols in parallel. Typically, the VA span estimates the number of items simultaneously identified within a single fixation in the global or partial report tasks (Lobier and Valdois 2015; Valdois et al. 2003; Ramamurthy et al. 2025). It has revealed successful at Age 5 to predict reading acquisition 1 year later. However, as it requires high‐level visual recognition and categorization of non‐alphabetic symbols, or verbal report of briefly presented letter or digit strings, its assessment at Age 3 is particularly challenging. Consequently, VA deficits that may impede reading acquisition are often identified later than phonological deficits.

We considered symbol‐based visual search to be an especially promising candidate for early detection of VA deficits, as it is both simple enough to be administered reliably by Age 3 and specifically engages several VA processes that have been implicated in DD: simultaneous multi‐element processing (Lobier et al. 2014; Peyrin et al. 2011, 2012; Ramamurthy et al. 2024;Vialatte et al. 2023a), visuo‐spatial perception of graphic symbols (Castles and Coltheart 1993; Marendaz et al. 1996; Franceschini et al. 2012; Bellocchi et al. 2017; Pisella et al. 2021; Vialatte et al. 2023a), as well as both overt (Vernet et al. 2022) and covert (Facoetti et al. 2010; Franceschini et al. 2012, 2022) attentional orienting (endogenous rather than exogenous as shown by Ramamurthy et al. 2024). Moreover, these tasks require processing at both local and global scales, another dimension in which atypicalities have been documented in DD (Franceschini et al. 2017a).

**FIGURE 1:**
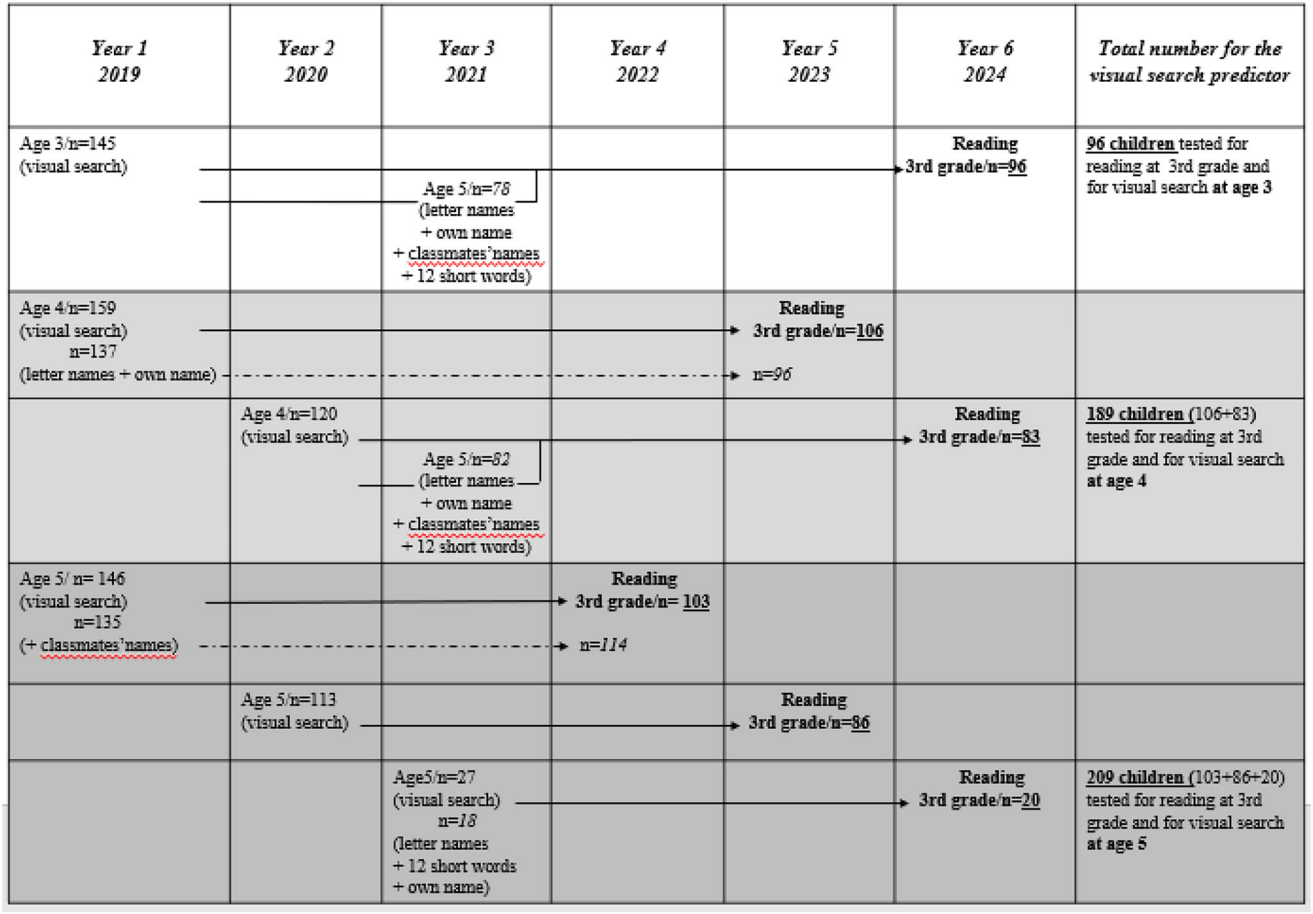
Year‐by‐year flow chart presenting the longitudinal study of the Ludisymboles project conducted in 11 French public schools of the Gex region over a period of 6 years.

In sub‐samples of the children tested longitudinally from Age 3, when feasible (see Figure 1), the Ludisymboles project additionally investigated other predictors of reading— administered at Ages 4 and 5 — such as the well‐established test of letter‐name knowledge (Foulin 2005; Ecalle et al. 2023; Macchi et al. 2024), as well as three tests frequently used by preschool teachers to evaluate the accuracy of visuo‐orthographic processing and the early ability of visual word form recognition. First, the recognition of the correct orthographic form of one's own name, presented alongside two distractor versions (modified by a single‐letter addition, deletion, or transposition), was chosen to evaluate the child's sensitivity to local orthographic changes within a globally familiar visual word form. This ability linked to fine‐grained visual‐orthographic processing may be important to distinguish between similar words during reading, Second, the recognition of classmates’ names was used to assess memory across exposures to orthographic patterns encountered frequently in the daily environment. Third, the recognition of 12 short and frequent words, which assesses early sight word reading and visual lexical access, could serve as a proxy for the ability to recognize whole‐word forms, a skill that may support children's transition from decoding to more fluent reading (Valdois et al. 2019b).

The relationship between the early VA screening test and these other predictors of later reading performance was also investigated to situate it among more proximal measures of pre‐literacy reading ability.

### 1.3 Rationale for Choosing Visual Search‐Based Assessment as Early Predictor of Reading and for the Gap Measure

Converging evidence links SPL‐mediated simultaneous visuo‐spatial processing with non‐phonological components of dyslexia (Kevan and Pammer 2009; Vidyasagar and Pammer 2010; Peyrin et al. 2011; Reilhac et al. 2013; Lobier et al. 2012, 2014; Lobier and Valdois 2015; Pisella et al. 2021; Valdois et al. 2019a; Liu et al. 2022). As reviewed in Vialatte et al. (2023a), the increased demands of simultaneous visual processing in written language provide the theoretical bases for the missing argument of specificity requested by Goswami (2015). Letter identification relies on the analysis of elementary separable visual features (Grainger et al. 2008; B et al. 2006; Seyll et al. 2020) with essential role of VA in their integration and in the differentiation of visually similar spatial configurations (Treisman and Gelade 1980; Palmer and Rock 1994; Carrasco 2011). Words are perceived only if each letter is above identification threshold (Pelli et al. 2003). Written languages are therefore characterized by a hierarchical nesting of elements (at least two levels: letters made up of multiple features, words made up of multiple letters). Each level requires a capacity of simultaneous visual processing to grasp initially unknown graphic sets. Therefore, while a reduced pool of VA resources would not preclude the simultaneous processing of multiple unitary objects, it would hinder the processing of multiple graphic symbols systematically encountered during reading, that specifically combines the demands of both spatial integration inter‐ and intra‐object.

The specificity of difficulties for the visual processing of multiple graphic symbols has been demonstrated in two visual search studies. The first study (Casco and Prunetti 1996) demonstrated that poor readers were slow only in visual search conditions involving shapes made up of the spatial combination of separable features (i.e., graphic symbols). A tilted bar presented among vertical bars was found as quickly as in typically developing readers but search was slower when this tilted bar was combined with other bars to form a multi‐featured shape (e.g., K or other non‐alphabetic symbol) among a set of Fs (or other non‐alphabetic symbols distractors). This impairment in symbol search also contrasted with the normal performance in searches defined by color, orientation, or even by the conjunction of the two, making it a difficult serial task. Importantly, this contrast precluded their common processes as explanation of poor reading, like general visual and motor processing speed, saccadic accuracy and exploration strategy, as well as executive functions. The second study (Khan et al. 2016) precisely showed the similar behavioral dissociation in a patient with chronic infraclinic simultanagnosia consecutive to bilateral SPL lesion: the selective slowing in symbols’ feature‐absent searches, contrasting with normal timing in the search of equivalent difficulty consisting to find a red disk among red squares and green disks (color and shape conjunction). Interestingly, this study showed that this behavioral visual search difference expressed in time reflected an impairment of spatial attention (see also Valdois et al. 2019a for the absence of impairment of temporal attention in this patient with bilateral SPL damage). Indeed, the slowness specific to symbols could be mimicked in control subjects by artificially applying a spatially‐reduced visibility window of search contingent to gaze. When the patient performed the symbol search with an artificially reduced gaze‐contingent window, this did not impair her performance, confirming that it was fitting her natural spatial reduction of VA when processing symbols (Khan et al. 2016). Further assessments in this patient showed impairment expressed as a reduced VA span of letters, typical of DD, in the global report task (Valdois et al. 2019a) and in search tasks involving multiple visual stimuli made up of separable strokes that can be spatially combined in unpredictable manners, like letters (Vialatte et al. 2021a, 2021b). In contrast, the simultaneous visual processing capacity was not reduced for color disks (Vialatte et al. 2021a, 2021b), as also previously shown in DD (Valdois et al. 2012).

Vialatte et al. (2023a) argued that the temporal Gap between these visual search conditions is a task‐difficulty asymmetry specific of spatial attention capacity: the processing of multiple graphic symbols multiplies the need of simultaneous visual processing compared to multiple single‐unit objects. In search of a marker of reading difficulty specific to a reduction of VA resources, Vialatte et al. (2023a) therefore measured the reaction time difference score between searches involving symbol versus single‐unit object in a group of children with DD. Those with a pathological reaction time difference compared to normal readers displayed a reduced attentional window of search specifically for symbols, demonstrated through the gaze‐contingent moving window paradigm, as in the patient with bilateral SPL lesion (Khan et al. 2016; Vialatte et al. 2021a). The difference between multiple visual processing of symbols versus single‐unit objects thus appeared relevant to spot the SPL‐dependent VA capacity for multi‐element processing, also characteristic of the reduced VA span in DD.

We therefore designed our ***visual search screening test*** in order to detect those children showing this reaction time difference. It was also aimed to be feasible as early as Age 3, and independent of phonological or literacy knowledge, ensuring that performance reflects VA ability rather than familiarity with linguistic material. It contrasted color‐based and symbol‐based search performance. The task required children to locate and touch a single black circle as quickly as possible, with half of the trials presenting the target among colored circles (color condition) and the other half among black circles each paired with a bar of varying length and spatial position (symbol condition). Trials from both conditions were randomly interleaved. The validation study (Vialatte et al. 2025) involved the assessment of 2015 children between 3 and 11 years old, providing age‐normative values of the Gap measure. Establishing that both the Gap and its variability decreased with age, the individual gaps of the 2015 children were plotted by continuous ages and fitted using the sum of an exponential and a linear function to compute age‐independent standardized Gap. Satisfactory test–retest reliability of this standardized measure was found on a sample of 69 individuals. The relationship between the Gap and the reading efficiency was assessed in 769 children of 4th and 5th grade. Reading efficiency was significantly correlated to the Gap, it predicted 5.3% of the variance of reading efficiency. The centile categorization of performance in the visual search screening test and in the reading test were not statistically different.

The results of Vialatte et al. (2025) therefore established the ability of the visual screening test and its Gap measure to detect the deficits of VA correlated to reading performance, based on the concurrent measures collected in a group of children aged between 8 and 11 years old. Building on these previous findings, we hypothesized that the visual screening test and its Gap measure could serve as an early specific marker of vulnerability, focusing solely on the reduction of VA capacity likely to compromise reading development. A large majority of the children of 3, 4, and 5 years of age involved in this previous validation study (Vialatte et al. 2025) later completed the reading assessment when they reached the third grade. The present study is the report and analysis of this longitudinal data set allowing us to evaluate the predictive validity of our VA screening tool and to identify the thresholds providing satisfactory sensitivity and specificity.

## 2 Methods

All experimental procedures were approved by the French health research ethics committee (CPP Ile de France VI, 2017‐A02525–48) and involved parental written informed consent.

### 2.1 Recruitment

**FIGURE 2:**
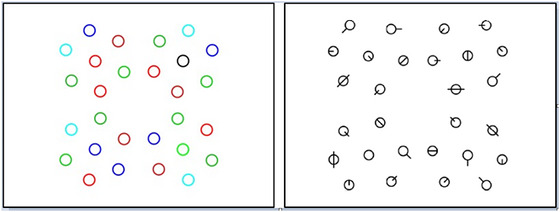
Examples of visual search displays. The unique visual target (the black circle) was presented among circles of various colors (left panel) versus among circles combined with a bar of various length and location (right panel).

A total sample of 710 children aged between 3 and 5 years (sex ratio = 1.01) was recruited from public kindergarten in the South Gex region (France) over a period of 3 years (2019‐2020‐2021, Figure 1). The data were collected across 11 schools located in urban, peri‐urban, and rural areas (Figure 2), mixing various socioeconomical status.

Among this total sample screened for visual search performance during the first 3 years of the project, a total of 137 four‐years‐old children and 162 five‐years‐old children, distributed across these areas, were also assessed for other potential or established predictors of reading (Letter names, own name, classmates’ names, 12 short words, Figure 1).

A subsample of 494 children were still scholarized in these 11 participating schools of south Gex region when they reached the third grade and could therefore receive an experimenter‐blind assessment of their reading performance in 2022–2023 or 2024 (Figure 1).

### 2.2 Evaluation of the Visual Screening Test as Early Reading Predictor

#### 2.2.1 The Visual Search Screening Test

The visual search screening test consisted of 64 visual search displays in which participants were instructed to find and touch the (unique) black circle as quickly as possible. The circle diameter was 1.6° at a viewing distance of 35 cm. The target (i.e., the black circle) was presented either among circles of various colors, or among black circles combined with a bar of various length and location (Figure 2). The target could appear equally often in the four quadrants and at four different eccentricities (10, 15, 20 and 25° from the center for 35 cm eye‐screen distance) with different inter‐item spacing. These variations aimed at avoiding habituation to the locations of the target and the 27 distractors across time, though minimizing visual crowding (the metrics kept the interspacing outside the critical distance specific to each visual eccentricity of the Bouma law).

For each trial, the software recorded the time at which the participant touched within a circular diameter of 3.7° visual angle centered on the target location. This touch ended the trial. Then, an inter‐trial display was presented, consisting of a centered black circle, to capture the participant's attention and remind the target to be found, before visual search display presentation.

The instructions were provided using example protocol images printed on paper sheets. The task was explained as follows: ‘You have to find this black circle (showing the circle alone on a page), which will either be among other colored circles or among other black circles, each of which are associated with a bar.’ The child was shown two trial examples as paper images (one with colored circles and one with symbols) and was asked to point toward the black circle on the laminated sheets. Once the experimenter ensured that the child understood this basic instruction by the child's pointing responses, the child was informed that they would perform in the same manner on the tablet until he finds all the 64 black circles hidden in the images, and that he has to be the fastest as possible. It was also mentioned that the black circle will be presented alone in‐between each search image, and that this display was simply to look at “to remind you that you should find this same black circle in all images”.

Children were seated on height‐adjustable stools to ensure a screen‐to‐eye distance of 35 cm, which was verified with a tape measure. The start signal was given, and the ‘Start’ button was pressed. The experimenter remained next to the child (on the right side) to ensure that they stayed focused throughout the test. If necessary, the experimenter could refocus and encourage the child. In cases where an event occurred during a trial leading to an unusually long pointing response time, those trials were excluded from the offline analyses.

Offline analyses of each individual participant's data consisted of extracting the median (Q50) and interquartile range (IQR) of the search times separately for color (c) and for symbol (s) trials. Trials with reaction times identified as outliers (i.e., exceeding the mean reaction time by more than 2.5 times its variance) were removed from further analyses. The individual Gap (G) was then computed as follows:

Since both the Gap and the Gap variability varied with age, a ***visual search percentile*** was then derived using the age norms published in Vialatte et al. (2025).

#### 2.2.2 The Alouette Reading Test

This reading task (Lefavrais 1967 ) evaluates lexical decoding under normal reading conditions using a text that is grammatically and syntactically correct, composed of real words but overall meaningless. This makes the predictability of content words very low. As a result, the Alouette Test does not provide contextual cues that the reader can use to compensate for decoding difficulties (Seyll et al. 2020). The child was informed that the text is meaningless. The instruction was to read the text as best as possible for a period of time of 3 min. If the child finished reading the text before the time limit, the experimenter recoded the exact duration required. The experimenter also collected the number of reading errors and the total number of words read. From these measures, the **reading inaccuracy** (or error rate) was computed as the number of errors divided by the total number of words read by the participant, and the **reading speed** as the number of words read by the participant divided by the reading time in seconds. Individual z‐scores were then computed as the standard deviation to the population means in speed and accuracy, separately. Then, the **z‐scores of reading efficiency** were calculated for each child as the mean of their speed and inaccuracy z‐scores.

#### 2.2.3 Demographic Information

Table 1 provides demographic information of the subsample of 494 children who received both the visual search screening test at 3, 4, or 5 years old and the Alouette reading test in 3rd grade.

**TABLE 1:** Demographic information and number of participants assessed for both the visual search screening test and the Alouette reading test.

Subsample assessed for visual search in kindergarten and reading at 3rd grade	Total number	Age (SD)	Sex ratio	Urban area	Suburban area	Rural area
**Visual search at Age 3**	96	3.8 (0.3)	1.46	43	34	19
**Visual search at Age 4**	189	4.6 (0.3)	1.08	44	69	76
**Visual search at Age 5**	209	5.6 (0.4)	0.9	36	84	89
**Total**	494	4.9 (0.8)	1.06	123	187	184

#### 2.2.4 Statistical Tests of the Relationship Between Reading and Visual Search

We first evaluated, on the full sample of 494 participants and separately for each kindergarten age group, the simple correlation between the individual visual search percentiles and the reading efficiency z‐scores. We expect a negative correlation such that children with the highest Gap in kindergarten will exhibit the lower reading performance at 3rd grade.

Second, we aimed at examining the relationship between visual search and reading using Chi‐square analyses. Based on the observed frequencies of children in each cell of the contingency table crossing two categorical variables, these analyses required to classify participants’ performance levels across variables.

We applied two predefined thresholds indicating fragility and pathology. A performance was considered fragile when it fell within the lowest‐performing 25% of the distribution, and pathological when it fell within the bottom 5%. For response time measures (the standardized Gap)—where longer durations reflect poorer performance—these thresholds correspond to the 75th and 95th percentiles, respectively. For accuracy‐based measures, in contrast, they correspond to the 25th and 5th percentiles. When variables followed a normal distribution, such as the reading efficiency z‐score, these thresholds were equivalent to *z* = –0.674 for fragility and *z* = –1.65 for pathology. These categorical variables provided the common framework to explore the association between early visuo‐attentional ability and subsequent reading outcome.

Using these criteria, we categorized both early visual performance and later reading ability. Visual search performance was classified according to age‐normed percentiles of the Gap: normal (<75th percentile), fragile (75th–95th percentile), and pathological (>95th percentile). Reading efficiency, as measured by z‐scores on the Alouette test, was grouped similarly: normal (*z* > –0.674), fragile (–1.65 < *z *≤ –0.674), and pathological (*z* ≤ –1.65). Normal/fragile/pathological observed counts were extracted in the full sample from the standardized Gap percentiles of the visual search screening test on one side, and from the *z*‐scores of reading efficiency at the Alouette test on the other side. Because Chi‐square analyses require a minimum of five individuals per cell, the three‐level classification (normal, fragile, pathological) could not be applied in age‐specific analyses. We therefore extracted only two categories from the standardized Gap percentiles with either the fragility (>75th percentile) or the pathology (>95th percentile) thresholds to ensure valid statistical testing. Significant Chi‐square results would indicate that the two categorical variables (visual attention profile and reading outcome) are statistically dependent, providing additional support for an association between early visual performance and later reading ability.

With the same categorization, we then assessed the sensitivity and specificity of the visual search screening test at each age (3, 4, or 5 years old) in detecting reading efficiency fragility. For these analyses, the visual search screening threshold was set at either the 75th or 95th percentile. We therefore tested the predictive value of the visual search screening test at each age using fragility or pathology thresholds, in relation to the risk of reading difficulties—including mild ones—defined by the fragility threshold on the Alouette reading test.

As a final step, odds ratios (OR) were computed to assess group differences in outcome likelihood across the predefined categories. An OR greater than 1 indicates higher odds of the outcome relative to the reference group, whereas an OR less than 1 indicates lower odds. These results provide a clearer interpretation of the strength and direction of the associations between performance levels and outcome measures.

### 2.3 Other Predictors

Among the subsample of 494 children who could be assessed for reading in 3rd grade, in addition to the visual screening test, 96 were assessed at 4 years old for letter names knowledge and own name recognition and 292 in total could be assessed at 5 years old for other predictors (Figure 1).

#### 2.3.1 Tasks and Measures

For the letter name knowledge assessment, the 26 letters were all presented in black upper case in a random order on an A4 sheet of paper. The child was requested to point and name each letter. The experimenter pointed to the unidentified items until 3 min elapsed. The number of correct identifications was scored (/26).

For the assessment of the recognition of one's own name, the child was presented with a row of three words written in black upper case and requested to point to « the one that corresponded exactly to their own first name ». The two distracter forms corresponded to the first name modified by a letter change, addition, deletion or inversion (e.g., MAROIN NARION MARION). The correct orthographic form was always the third. The score was 1 if correct, 0 if incorrect.

For the recognition of classmate's names, the first names of 19 classmates were presented in black upper case together with their own first name on the same A4 sheet of paper. The child was requested to point and name all the first names they could recognize. The experimenter pointed to the unidentified items until 3 min elapsed. The number of correct identifications was recorded (/20).

For the recognition of short and frequent words, the child was requested to point and recognize 12 French words (selected by Valdois et al. 2019b) written in black upper case and presented in two columns on a A4 sheet of paper (LE, LA, UNE, OU, RI, LU, SON, AMI, ECOLE, PAPA, MAMAN, NOËL) (respectively: the (male), the (female), a (female), or, laughed, read, his, friend, school, daddy, mummy, Christmas). All were short frequent words that usually occurred on posters in classrooms or in children's picture books (mean frequency in first grade books = 8006 per one million, from MANULEX: Lété et al. 2004). The experimenter pointed to the unidentified items until 3 min elapsed. The number of correct identifications was recorded (/12).

#### 2.3.2 Statistical Tests of the Relationship Between Reading and Other Predictors

Correlations were separately assessed, with smaller samples, between reading performance at 3rd grade and the different predictors tested at 4 and 5 years old, except for the recognition of one's own name because it was a binary variable. This latter measure was collected at Ages 4 and 5, and we tested its association with reading performance in 3rd grade as assessed by the Alouette test in two ways. First, Student's *t*‐tests were used to compare reading efficiency scores between children who correctly identified their name in preschool and those who did not. Additionally, Chi‐square analyses were conducted to examine whether children who failed to recognize their name were more frequently categorized as fragile readers in third grade.

### 2.4 Statistical Tests of the Relationship Between the Visual Search Screening Test and the Other Predictors

We further examined the relationship between the visual search performance at 3, 4, or 5 years old and the other predictors assessed as a reflecting « early familiarity with written language ».

#### 2.4.1 Relationship Between the Standardized Gap and the Ability to Recognize One's Own Name

This relationship was assessed by comparing the standardized Gap values according to whether children correctly recognized their own first name in the final year of kindergarten. A Student's *t*‐test was used to compare mean Gap values between these two groups of children, and using a Chi‐square test on the previously defined two categories of visual test performance level with the thresholds of either fragility (25th percentile) or pathology (5th percentile). These analyses included all children performing the own name recognition test at 5 years old and the visual search screening test at 3 (*n* = 78), 4 (*n* = 82), or 5 (*n* = 18) years old, hence a total of 178 children (see Figure 1).

#### 2.4.2 Relationship Between the Standardized Gap and the Ability to Benefit From Repeated Expositions to Written Material (Classmates’ Names and 12 Short and Frequent Words)

This was studied for each of the other written language tasks assessed at 5 years old (recognition of classmates’ names and of 12 short and frequent words) by studying their correlations with the standardized Gap value obtained at either 3, 4, or 5 years old.

For the test of the classmates’ names recognition, this could be run on a total sample of 295 children who were all evaluated in the last year of kindergarten (at about 5 years old) and performed the visual search screening test either the first year of kindergarten (at about 3 years old, *n* = 78), the second one (at about 4 years old, *n* = 82) or the third one (at about 5 years old, *n* = 135).

For the test of the 12 short and frequent words, analyses were conducted on a total sample of 178 children who were all evaluated in the last year of kindergarten (at about 5 years old) and performed the visual search screening test either the first year of kindergarten (at about 3 years old, *n* = 78), the second one (at about 4 years old, *n* = 82) or the third one (at about 5 years old, *n* = 18).

#### 2.4.3 Relationship Between the Standardized Gap and the Knowledge of Letter Names

This was studied for the recognition of letter names assessed either at 4 or at 5 years old by studying their correlations with the standardized Gap value. This could be run on a total sample of 178 children who were all evaluated for letter names in the last year of kindergarten (at about 5 years old) and performed the visual search screening test either the first year of kindergarten (at about 3 years old, *n* = 78), the second one (at about 4 years old, *n* = 82) or the third one (at about 5 years old, *n* = 18).

This could also be run on a total sample of 137 children who were all evaluated for letter names and visual search in the second year of kindergarten (at about 4 years old when letter names knowledge is more strongly associated with future reading performance) on Year 1 (see Figure 1).

## 3 Results

### 3.1 Evaluation of the Visual Screening Test as Early Reading Predictor

#### 3.1.1 Correlations Between Percentile at the Visual Search Screening Test Performed in Kindergarten and Z‐Score of 3rd Grade Reading Efficiency

**FIGURE 3:**
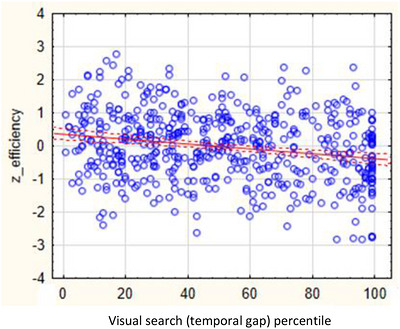
Significant correlation between reading efficiency tested in third grade and Visual search (Gap) percentile in the whole sample tested in kindergarten.

Figure 3 displays the correlation for the full sample (gathering children assessed at 3, 4, and 5 years old and categorized in percentiles) which appeared highly significant (r = −0.24, p < 10−6, n = 494).

**FIGURE 4:**
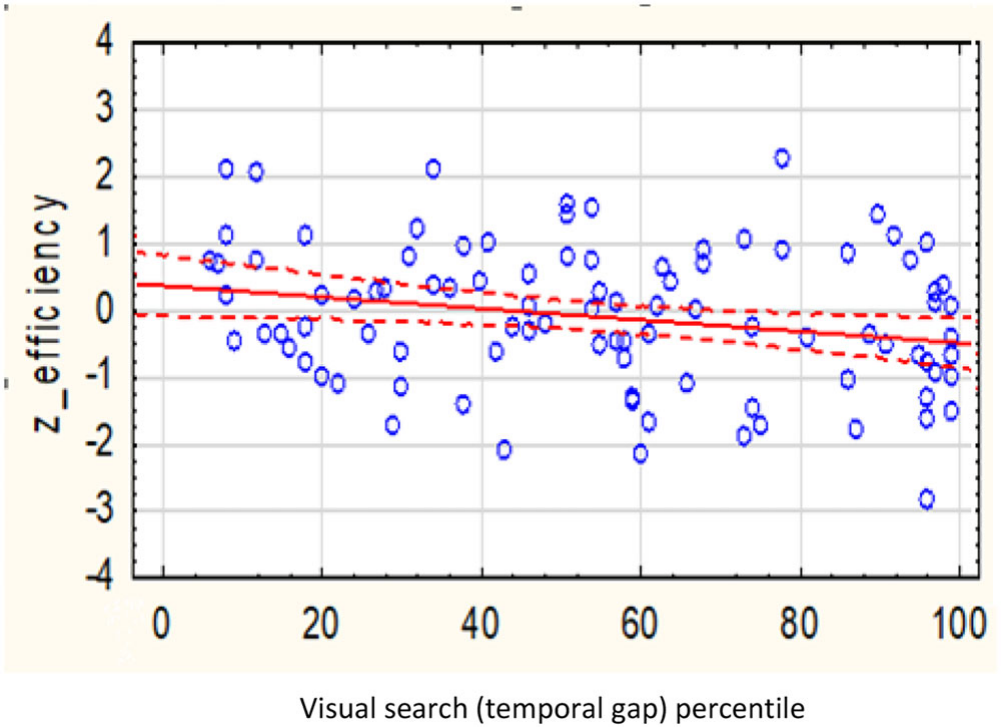
Significant correlation between reading efficiency tested in third grade and Visual search (Gap) percentile tested at age 3.

The correlations performed separately when the visual search screening test was run at 3 years old (r = −0.24, p = 0.020, n = 96), at 4 years old (r = −0.21, p = 0.003, n = 189) and at 5 years old (r = −0.26, p = 0.00015, n = 209) were also significant and displayed similar coefficients between each other and also compared to the full sample. As an example, Figure 4 illustrates the correlation for children assessed at 3 years old.

#### 3.1.2 Comparison Between Centile Categorization of Performance in the Visual Search Screening Test Performed in Kindergarten and in the 3rd Grade Reading Test

Another way to test the relationship between the visual search test and reading performance was to statistically compare the hierarchical centile position of children along these variables. Altogether, when assessed in kindergarten, the Gap centile position did not differ to the reading one.

For the full sample, when the normal/fragile/pathological categorization was compared, 𝜒^2^ tests demonstrated that visual search screening and later reading efficiency were not independent (*χ* ^2^ (4) = 34.619, *p* < 0.001)

For each age category, *χ* ^2^ tests demonstrated that categorization of visual search and reading performance with the threshold of the worse 25% were not independent whether the Gap percentile was assessed at 3 years old (*χ* ^2^(1) = 4.5827, *p* < 0.05), at 4 years old (*χ* ^2^(1) =  11,695, *p* < 0.001) or at 5 years old (*χ* ^2^(1) =  7.1678, *p* < 0.01).

For each age group, with the pathological threshold (95th percentile) defined for the visual search screening test and the fragile threshold (*z*‐score ≤ –0.674) defined for reading efficiency, *χ* ^2^ tests revealed that visual search performance and reading efficiency were not independent when the Gap percentile was assessed at Age 3 (*χ* ^2^(1) = 8.48, *p* < 0.01) and at Age 4 (*χ* ^2^(1) = 12.17, *p* < 0.001). However, at Age 5, no significant association was observed between the two measures (*χ* ^2^(1) = 2.41, *p* = 0.12).

Contingency tables are provided as supplementary information.

#### 3.1.3 Specificity and Sensitivity of the Visual Search Screening Test

We also evaluated the specificity and sensitivity of the visual search screening test in detecting reading difficulties with the threshold clinically defined as reading efficiency <25th centile (*z*‐score below –0.674). Note that, given that the base rate of reading fragility in our sample was 26%, it is not biased and rather fairly representative.

Sensitivity values were expected to be relatively low, as a substantial proportion of the sample may exhibit reading difficulties unrelated to the specific visuo‐attentional processes targeted by our screening tool. For instance, false negatives (FNs)—children showing normal visual search performance but later experiencing reading difficulties—are likely in cases of primarily phonological deficits that hinder reading acquisition.

In our context, achieving high specificity is of greater importance, to ensure that children identified as at risk genuinely present a visuo‐attentional profile warranting early intervention. False positives (FPs)—that is, children exhibiting fragile or impaired performance on visual search tasks without subsequently developing reading difficulties—represent a limitation in diagnostic approaches, where specificity is critical. However, within a screening framework, their occurrence is less concerning, since the primary goal is to identify at‐risk children who may benefit from further clinical assessment and early training aimed at strengthening visual‐attentional prerequisites essential for reading acquisition.

With the full sample of children tested for visual search in kindergarten, the false positive rate was 54% with the threshold set at the 95th percentile (with 77% true negatives, yielding 24% sensitivity and 93% specificity), and 44% with the threshold set at the 75th percentile (with 80% true negatives, yielding 44% sensitivity and 80% specificity).

The results were even more robust when considering only the subsample of children tested at Age 3, with the visual search threshold set at the 95th percentile. Sensitivity reached 36% and specificity 94%. Among the 16 children who tested positive on the visual search task, 10 later showed reading difficulties (true positive rate: 62.5%), whereas among the 80 children who tested negative, only 18 subsequently exhibited reading difficulties (false negative rate: 22.5%). When the visual search threshold was lowered to the 75th percentile, sensitivity increased to 46%, while specificity decreased to 78% at Age 3.

For the sample of 4‐year‐old children, sensitivity was 25% and specificity 94% when the threshold was set at the 95th percentile, while sensitivity increased to 42% and specificity decreased to 84% with the threshold set at the 75th percentile.

For children tested at Age 5, the screening test did not perform as well as at earlier ages (95th percentile: 16% sensitivity, 92% specificity; 75th percentile: 44% sensitivity, 76% specificity).

#### 3.1.4 Odds Ratios

Another way to interpret our results was through odds ratios (ORs).

At Age 3, with the visual search threshold set at the 95th percentile, the OR was 5.74, indicating that children who failed the visual search task were 5.7 times more likely to exhibit reading difficulties in 3rd grade. When the threshold was lowered to the 75th percentile, the OR decreased to 3.10.

At Age 4, the results were similar. With the threshold set at the 95th percentile, the OR was 5.54, indicating that children who failed the visual search task were 5.5 times more likely to experience later reading difficulties. With the 75th percentile threshold, the OR was 3.70.

When the visual search screening test was administered at Age 5, ORs were lower. With the 95th percentile threshold, the OR was 2.30, and with the 75th percentile threshold, 2.53. This suggests that the predictive relationship between early visual search performance and later reading difficulties decreased with age.

### 3.2 Other Preschool Predictors of 3rd Grade Reading Efficiency

#### 3.2.1 Correlations Between Other Predictors Assessed at Ages 4 or 5 and Z‐Scores of 3rd Grade Reading Efficiency

**FIGURE 5:**
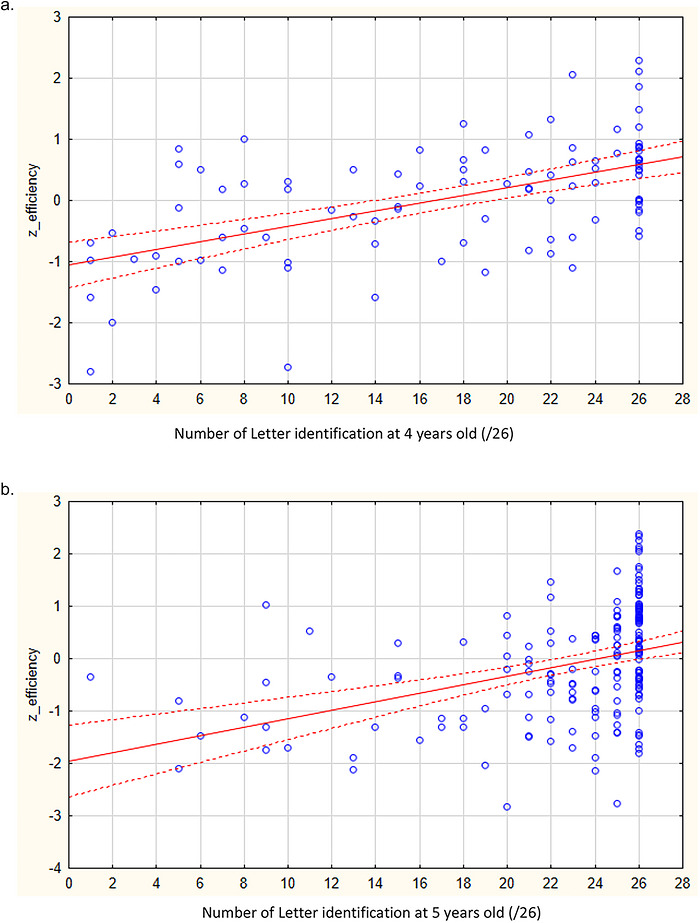
Significant correlation between reading efficiency tested in third grade and (a) Number of letter identification at 4 years old (/26), (b) Number of letter identification at 5 years old (/26).

For the letter names knowledge, the correlation was highly significant, with higher coefficient when it was evaluated at 4 years old (*r* = 0.55, *p* = 2.2 × 10^−10^, *n* = 96, Figure 5a) than at 5 years old (*r* = 0.38, *p* = 1.34 × 10^−7^, *n* = 178, Figure 5b) because of a visible ceiling effect.

**FIGURE 6:**
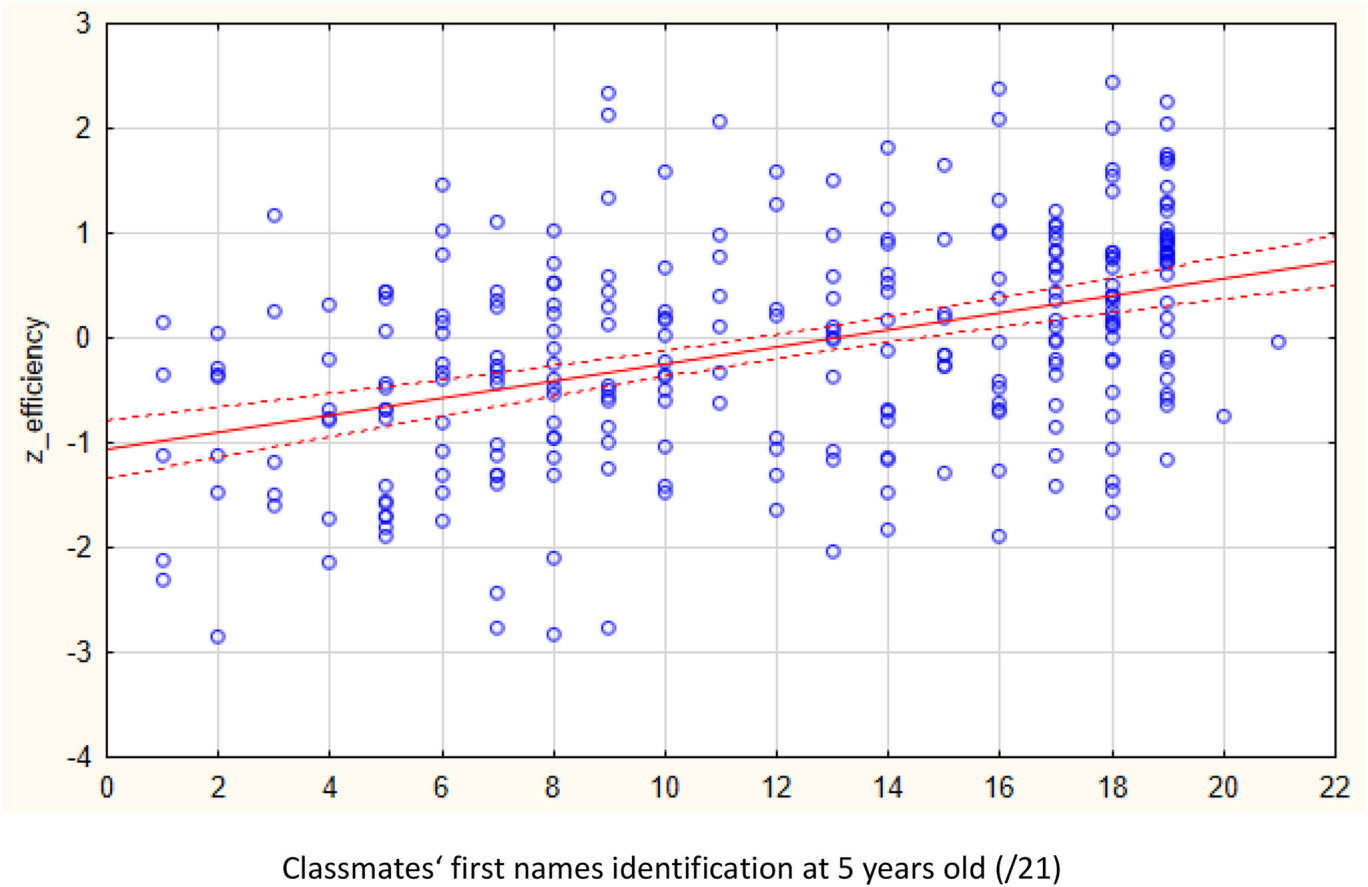
Significant correlation between reading efficiency tested in third grade and Classmates’ first names identification at 5 years old (/21).

For the recognition of the classmates’ first names proposed to children at 5 years old, the correlation was highly significant (*r* = 0.42, *p* < 10^−6^, *n* = 274, Figure 6)

**FIGURE 7:**
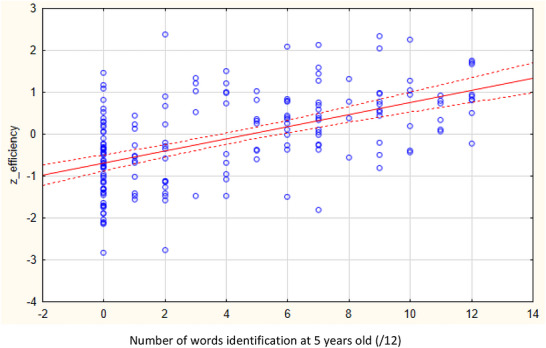
Significant correlation between reading efficiency tested in third grade and Number of words identification at 5 years old (/12).

For the recognition of 12 short and frequent words proposed to children at 5 years old, the correlation was highly significant (*r* = 0.54, *p* = 8.1 × 10^−15^, *n* = 177, Figure 7)

In summary, the closer the preschool predictor is to the onset of reading, the later it becomes measurable, but the stronger its correlation with the Alouette reading test in 3rd grade.

#### 3.2.2 Relationship Between Ability to Recognize One's Own Name at Ages 4 or 5 and Z‐Score of 3rd Grade Reading Efficiency

The strength of correlation could not be compared for the predictor evaluating the ability to recognize one's own name, a binary measure. Instead, we sought to determine whether this measure, assessed at 4 or 5 years old, was predictive of reading performance below fragility threshold.

At Age 4, children who correctly recognized their own first name obtained significantly higher reading efficiency scores in third grade, as measured by the Alouette test (*t*(94) = −2.78, *p* = 0.008). Furthermore, Chi‐square analyses revealed a significant association between early name recognition and later reading status, with children who failed to recognize their name more frequently classified as fragile or pathological readers (*χ* ^2^(1) = 7.8, *p* = 0.005). These findings support the predictive value of name recognition at this early stage for later reading performance.

At Age 5, the one‐tailed Student's *t*‐test revealed a slight but significant improvement in third‐grade reading efficiency scores among children who correctly recognized their own name, (*t*(176) = −1.78, *p* = 0.038) compared to those who did not. However, the Chi‐square test did not reach significance (*χ* ^2^(1) = 0.02, *p* = 0.872), suggesting that this difference in reading scores was not substantial enough to shift the distribution of children across categorical reading outcomes (normal vs. fragile/pathological).

### 3.3 Relationship Between the Visual Search Screening Test and the Other Reading Predictors

#### 3.3.1 Relationship Between the Standardized Gap and the Ability to Recognize the Exact Orthography of One's Own Name

**FIGURE 8:**
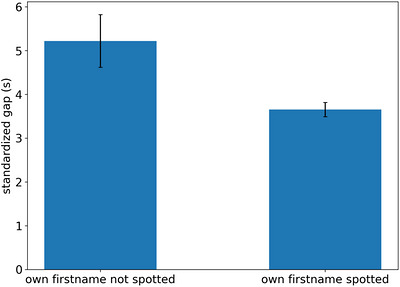
Mean and SD of the standardized gap measure extracted from the visual search performance in the group of children who correctly identified their own name at 5 years old versus those who did not.

For the 178 children who performed the test of own name's recognition in the last year of kindergarten (at about 5 years old), the standardized Gap categorization significantly predicted whether the child was able to spot his/her own name among orthographic neighbors. This was demonstrated with the threshold of the 95th percentile (*χ* ^2^(1) = 7.4, *p* = 0.006), as well as with a *t*‐test establishing that the standardized Gap was significantly different between children who correctly spotted their own name and children who did not (Figure 8; *t*(176) = 3.55, *p* = 0.0005). The mean standardized Gap in the group of children who did not spot their own name at 5 years old was 5.22 sec (Figure 8), which corresponds to a performance at the 85th percentile on the visual search screening test.

#### 3.3.2 Relationship Between the Standardized Gap and the Ability to Benefit From Repeated Expositions to Written Material (Recognition of Classmates’ Names and 12 Short and Frequent Words)

The standardized gap was significantly correlated with both tests of early sight word recognition: the recognition of 12 short and frequent words (*r*(176) = −0.12, *p* = 0.048) and the recognition of classmates’ names (*r*(293) = −0.26, *p* = 3.9 × 10^−15^).

#### 3.3.3 Relationship Between the Standardized Gap and the Knowledge of Letter Names

The standardized gap was not significantly correlated with the letter‐name knowledge test administered in the last year of kindergarten (at approximately 5 years of age, *r*(176) = –0.11, *p* = 0.14), nor in the second‐to‐last year of kindergarten (at approximately 4 years of age, *r*(135) = –0.05, *p* = 0.589).

## 4 Discussion

Most research on early reading acquisition has emphasized the role of phonological processing, and oral language in general, in the development of decoding skills (Scarborough 2001; Vellutino et al. 2004; Ehri 2005). However, phonological variability alone does not fully account for the heterogeneity of children's reading and spelling outcomes (Cunningham et al. 2002; Bosse and Valdois 2009, Perry and Long 2022). Several studies measuring pre‐reading VA ability at 5 years old, and testing reading abilities at 6 years old in first grade, have put forward VA parameters as good predictors at the end of kindergarten of reading acquisition one year later (Pisella et al. 2021; Bosse et al. 2009; Franceschini et al. 2012; Valdois et al. 2019b; Saydam and Bastug 2021; Vernet et al. 2022; Ramamurthy et al. 2025). This suggests that learning to read implies not only good language skills, but also adequate VA prerequisites. However, these VA predictors remained quite proximal to reading ability, in the sense that they either require digit or letter knowledge or simulate some visual aspects of reading like left‐to‐right and global‐to‐local sampling of graphic symbols displayed horizontally (similar to words or lines of text). It is thus not excluded that reduced abilities with such visual displays, even measured at 5 years old, were already secondary consequences of non‐VA disorders and reduced literacy rather than causally linked to reading acquisition. Accordingly, the strength of these predictors tends to increase with age and print exposure (Vernet et al. 2022, 2023).

Other studies have demonstrated that VA training can transfer to reading improvement in DD (Antzaka et al. 2017; Franceschini et al. 2017b; Vialatte et al. 2023b). Theoretically, such interventional studies provide additional arguments in favor of the causal contribution of VA deficits to some forms of DD (Lobier and Valdois 2015). Practically, they also increase the relevance of detecting VA deficits and run specific VA training programs as early as possible to maximize their efficacy. To these respects, detecting VA deficits prone to hinder reading acquisition as early as Age 3 was a relevant challenge both theoretically and practically. Oral language predictors already exist to screen for the phonological deficits at Age 3 and help to reduce the delay in reading acquisition and its consequences on self‐esteem and behavior at school thanks to early speech‐therapy interventions. A complementary test able to screen so early for the VA aspects of reading was lacking. In such circumstances, we needed a measure able to identify a small high‐risk subgroup with specific VA deficits rather than functioning as a broadly sensitive early screening instrument to predict reading difficulties. Such high specificity requires tasks and visual displays more distal from reading than those of predicters previously validated for the end of kindergarten (Bosse et al. 2009; Franceschini et al. 2012; Valdois et al. 2019b; Saydam and Bastug 2021; Vernet et al. 2022; Ramamurthy et al. 2025), even if the purer the measure, the smaller the correlation strength and the less sensitivity to predict multifactorial reading difficulties. Visual search tasks involving non‐verbal graphic symbols not displayed in horizontal lines appeared not only feasible at Age 3 but also ideal to investigate such selective VA skills independent from print experience. Previous results suggested that a specific slowness of search for a target distinguished by a missing element in a graphic symbol is linked to poor reading performance (Casco and Prunetti 1996; Marendaz et al. 1996; Vialatte et al. 2023a, 2025). Such a specific visual search deficit has been associated with the superior parietal lobules (SPL) based on lesion studies (Khan et al. 2016; Vialatte et al. 2021a). Moreover, the SPL has been put forward by functional neuroimaging as underlying substrate bilaterally and specifically activated when several graphic symbols must be processed simultaneously (Lobier et al. 2012). Good capacity for simultaneous visual processing (VA span) is necessary to accurately apprehend the relative length, orientation and location of the different visual components of letters, and later the relative positions of multiple letters for word decoding. We therefore aimed at measuring the Gap between symbols and single‐unit objects as a SPL‐specific marker of VA linked to reading performance. Although most of these previous studies contrasted symbols with filled objects (Casco and Prunetti 1996; Khan et al. 2016; Vialatte et al. 2021a, 2023a), our visual screening test contrasted a symbol‐based feature absent search and a color‐based search allowing the instruction to be as simple as « always pointing the black circle ». This ensured its feasibility at Age 3. Vialatte et al. (2025) also confirmed the test‐retest reliability of the Gap centile categorization independent on age, and its relationship with concurrent measure of reading efficiency at Ages 9 or 10. As expected because reading performance is multifactorial, the correlation between the two measures was small (explaining 5.3% of the variance of reading efficiency) but significant. As expected because DD is not solely associated with VA deficit, the sensitivity of the Gap to detect the reading difficulties was moderate, but its observed specificity was high. Based on these previous promising results, we used the Gap in the present 6‐year longitudinal study, where the same children were administered the visual search test at Age 3 and the reading test when they reached the second semester of the 3rd grade. As discussed below, the predictive values of the Gap were satisfactory to detect a subgroup of high‐risk children who would benefit from specific and early VA training and who would probably not have been detected with the solely oral language predictors existing at Age 3.

First, the visual search screening test run at Age 3 predicted later text reading efficiency evaluated at Age 8 with similar correlation strength (*r* = −0.24, hence explains 6% of the variance of reading efficiency) as when it was run at Ages 9 and 10. Moreover, the correlation coefficients were not only comparable to those of the previous concurrent visual search and reading data collection (Vialatte et al. 2025), but they were also comparable whether the visual search screening test was run at 3, 4, or 5 years old during kindergarten. This modest but significant and stable correlation is the marker of a predictor of VA reading difficulty that is distal from reading and independent on print exposure. From our point of view, this consistent longitudinal associations may reveal a causal specific contribution to visual form of reading difficulty. However, the data remained correlational.

The value of the visual search screening test to detect future reading difficulty as early as Age 3 was confirmed by additional analyses comparing the distribution of the longitudinal sample across performance categories for both variables. When both visual and reading efficiency were set at fragility thresholds (75th percentile), the visual search screening test measure at Age 3 obtained specificity of 78% and sensitivity of 46%. As typical trade‐off, when the visual search threshold was increased to pathological performance, the Gap centile at Age 3 predicted fragile reading efficiency at 3rd grade with 94% specificity and 36% sensitivity. This allows for the early identification of children whose future reading could benefit from visuo‐spatial and visuo‐attentional training activities ahead of formal literacy instruction with true positive rate of 62.5%. Given the limited consequences for the few false positives of following training that would not be worth it, this approach remains appropriate for large‐scale screening. The odds ratios were favorable. Pathological visual search performance at Age 3 was associated with a 5.7‐fold increase in the likelihood of future reading difficulties. Note that missing 22.5% of the later fragile readers (true false negative rate) was also not disappointing as we did not expect a high sensitivity for our VA screening tool. Indeed, children with a good performance in the visual search screening test could still present reading difficulties due to pathological phonological awareness for example, and thus constitute false negatives. At 3 years old, the visual search screening test could therefore serve as a valuable complement to well‐established predictors of reading based on oral language (e.g., Scarborough 2001).

The Gap measure being a difference between two visual search conditions, it is considered free of all the common processes inherent to visual search, like general sensory and motor processing speed, general attentional stability, perceptual load linked to set‐size and item spacing, oculo‐motor processes and eye‐hand coordination to provide the manual response on the tactile screen. The difficulty asymmetry between the two search conditions was precisely what we intended to assess, arguing that the symbol condition involves more SPL‐specific VA resources of simultaneous visual processing because it combines both visuo‐spatial integration inter and intra‐item (Lobier et al. 2012; Khan et al. 2016; Vialatte et al. 2021a, 2023a). We argued that by multiplying the needs for simultaneous visual processing the symbol search condition will lead a child with reduced VA resources of simultaneous processing to enter in a more serial mode of processing, which is captured by both the search time median and variability differences (Gap measure) between the symbol and the baseline single‐unit object conditions. Unlike previous studies (Casco and Prunetti 1996; Khan et al. 2016; Vialatte et al. 2023a), the baseline condition of the visual search screening test is not a conjunction search. It is a color‐based search which may be less serial than the symbol‐based condition, even if we used gradual colors including the dark blue which was very close to the black target. As we did not vary the set size, we can only postulate that this color‐based condition does not pop‐out for young children of kindergarten, based on previous findings from our group (Vialatte et al. 2021a). Indeed, even in adults, such color‐based search involving several gradual colors was not fully pop‐out, as it was not free of significant set‐size effect and the gaze‐contingent moving window paradigm demonstrated that not all objects of the visual display could be processed simultaneously at initial central fixation. However, we acknowledge that the Gap may not solely measure SPL‐mediated VA, the symbol search condition may also require more executive control demands such as sustained attention and motivation to maintain the task objective as it is more difficult, and better exploration strategy as it is more serial.

Besides, we also aimed at testing in kindergarten new predictors assessing the ability to benefit from print exposure and the ease to memorize sets of graphic symbols. Like other important visual predictors (Bosse et al. 2009; Franceschini et al. 2012; Valdois et al. 2019b; Saydam and Bastug 2021; Vernet et al. 2022; Ramamurthy et al. 2025) more proximal to reading than the visual search screening test, they present the disadvantage of being only testable at 4 and 5 years old but showed stronger prediction strength.

Letter names knowledge confirmed its well‐established robustness (Foulin 2005; Ecalle et al. 2023; Macchi et al. 2024) to predict future reading performance in our sample at Age 4 (*r* = 0.55). However, when tested at 5 years old, it revealed less strong (*r* = 0.38) than the other tested predicters. For instance, in this last year of kindergarten, the more proximal measures evaluating the ability to recognize classmates’ names (*r* = 0.42) and 12 frequent words (*r* = 0.54) showed the highest correlation coefficients, as they were most directly related to reading.

Similarly to letter names knowledge, the visual recognition of one's own name among orthographic neighbors revealed significant predictor at Age 4, more strongly than at Age 5 due to a ceiling effect. This ability to differentiate the correct orthography of one's own name may be related to the ability to write one's own name, a predictor that previously emerged from the National Early Literacy Panel's (2008) meta‐analysis.

Interestingly, the Gap centile of the visual search screening test (performed at 3, 4, or 5 years old) significantly predicted the visual recognition at Age 5 of one's own name, of classmates’ names and of 12 short and frequent words. In contrast, the absence of a significant correlation between the Gap and letter‐name knowledge might reflect that alphabetical knowledge is rather shaped by culture, education, and phonological ability. It can thus be proposed that the Gap evaluates the VA capacity necessary to easily deal with printed words and memorize them, implicitly or explicitly. For the same reasons, we can also speculate that the Gap reflects the VA capacity that would be crucial for the switch from decoding procedure into fluent reading that typically occurs at the second semester of the 3rd grade (Aghababian and Nazir 2000; Chall 1983; LaBerge and Samuels 1974). These observed relationships with early word recognition further contribute to the validation of the visual search screening test as early predictor of reading. A limitation of this secondary aspect of our study is that it was designed for a single correlation analysis across time (longitudinal study) and the other tests were added in the meanwhile, when possible, without optimal statistical planification that would have allowed us to undertake multivariate analyses.

To conclude, it could be useful that the academic or health care prevention programs administer this simple screening tool. The visual search screening test demonstrated good specificity and favorable odds ratios for the detection, as early as Age 3, of a sub‐group of children at risk for future VA‐related reading difficulties. This would allow for personalized training that can be handled healthcare providers throughout the years of kindergarten to prevent their reading difficulties (e.g., computer games increasing their ability to simultaneously process spatial features of graphic symbols, Vialatte et al. 2023b) but also by pre‐school teachers using universal educational activities specific to visuo‐attentional processes aimed at optimizing the processing of letter strings. This can include oriented puzzles of letters, to get rid of visual confusions of similar forms and to allow letter to more quickly acquire the status of a well‐known single‐unit visual object and start earlier their parallel processing, and puzzles of words to deploy as early as possible specific attention on multiple letters and their order. For a better sensitivity and effective prevention, the visual search screening test should be combined with classical oral‐language predictors available at 3 years old to put more priority either on oral language or on VA abilities specific to written language. It should be completed when possible, later in kindergarten, by predictors more proximal to reading, like letter name knowledge and early word recognition, to confirm the needs of the children.

## Author Contributions

**Audrey Vialatte**: conceptualization, image designer, investigation, methodology, formal analysis, funding acquisition, writing – review and editing. **Eric Chabanat**: conceptualization, investigation, writing – review and editing, formal analysis, methodology. **Pierre–Emmanuel Aguera**: conceptualization. **Agnes Witko**: investigation, writing – review and editing, methodology. **Laure Pisella**: conceptualization, investigation, writing – original draft, funding acquisition, writing – review and editing, methodology, formal analysis, supervision.

## Funding

Funding for data collection was partly provided by Pulsalys grant R21036CC, AV received a grant from Fondation de France.

## Ethics Statement

All experimental procedures were approved by the French health research ethics committee (CPP Ile de France VI, 2017‐A02525–48).

## Conflicts of Interest

Audrey Vialatte is the co‐founder of the company Vialyy which develops and offers the task described in the present article following the positive results obtained.

## Supporting information




**Supporting Information**: desc70258‐supp‐0001‐SuppMat.docx

## Data Availability

The data that have been used are confidential
