# Supplementary material for: From Visual Attention to Literacy: Symbol Search Deficit Predicts Future Reading Difficulties From Age 3
Source: Dev Sci. 2026 Jul 22;29(5):e70258. doi: 10.1111/desc.70258 (PMC13392321; doi:10.1111/desc.70258)
Supplement: Supplementary file 1 — Supporting Information: desc70258‐supp‐0001‐SuppMat.docx [file DESC-29-e70258-s001.docx]

Contingency table showing the relationship between visual test performance in kindergarten and reading efficiency in Grade 3. The “fragile” group corresponds to the 25th percentile, and the “pathological” group corresponds to the 5th percentile.

For age 3

|  |  | reading efficiency | |  |
| --- | --- | --- | --- | --- |
|  |  | Normal | Fragile | All |
| visual search | Normal | 53 | 15 | 68 |
|  | Fragile | 15 | 13 | 28 |
|  | All | 68 | 28 |  |
|  |  |  |  |  |
|  |  |  |  |  |
|  |  | reading efficiency | |  |
|  |  | Normal | Fragile | All |
| visual search | Normal | 62 | 18 | 80 |
|  | Patho | 6 | 10 | 16 |
|  | All | 68 | 28 |  |

For age 4

|  |  |  |  |  |
| --- | --- | --- | --- | --- |
|  |  | reading efficiency | |  |
|  |  | Normal | Fragile | All |
| visual search | Normal | 118 | 28 | 146 |
|  | Fragile | 23 | 13 | 36 |
|  | All | 141 | 41 |  |
|  |  |  |  |  |
|  |  |  |  |  |
|  |  | reading efficiency | |  |
|  |  | Fragile | Normal | All |
| visual search | Normal | 133 | 36 | 169 |
|  | Fragile | 8 | 12 | 20 |
|  | All | 141 | 48 |  |

For age 5

| z |  | reading efficiency | |  |  |
| --- | --- | --- | --- | --- | --- |
|  |  | Normal | Fragile | All |  |
| visual search | Normal | 118 | 31 | 149 |  |
|  | Normal | 36 | 24 | 60 |  |
|  | All | 154 | 55 |  |  |
|  |  |  |  |  |  |
|  |  |  |  |  |  |
|  |  | reading efficiency | |  |  |
|  |  | Fragile | Normal | All |  |
| visual search | Patho | 142 | 46 | 188 |  |
|  | Normal | 12 | 9 | 21 |  |
|  | All | 154 | 55 |  |  |
|  |  |  |  |  |  |
|  |  |  |  |  |  |

For all children

|  |  | reading efficiency | | |  |
| --- | --- | --- | --- | --- | --- |
|  |  | Normal | Fragile | Patho | All |
| visual search | Normal | 289 | 62 | 12 | 363 |
|  | Fragile | 48 | 19 | 7 | 74 |
|  | Patho | 26 | 26 | 5 | 57 |
|  | All | 363 | 107 | 24 | 494 |
